# The Norfolk and Norwich Hospital, 1771-1847

**Published:** 1916-05-06

**Authors:** 


### SIDELIGHTS ON HOSPITAL HISTORY.

### The Norfolk and Norwich Hospital 1771–1847.

The following are portions of an interesting speech by Mr. E. B. Southwell, Lord Mayor of Norwich, at the annual meeting of the Norfolk and Norwich Hospital, as reported by the *Norfolk News*.

### Various Items in the Old Accounts.

In March 1771 William Pitt quotes for anchor and chimney irons at 3¼d. per lb., and Nicholas Frary acknowledges the receipt of 7s. 6d. for "chimneys rumaged down at Orspital at 6d. per chimney." In November 1772 T. Page accepts an order for a hospital clock "with a good sounding bell." These old letters indicate there was a considerable amount of trouble over contracts in connection with the building and extension of the hospital. John Green, who had authority to burn four clamps of 25,000 bricks on the ground, protests to the governors against a Mr. Blog being instructed to build the then remaining wing, whilst John Fox, having provided glass for the immediate furnishing of the newly-erected wing, protests against being put into competition with a price lower than his own by one penny per square foot, and justified his own figure by imputing indifferent workmanship to his opponent, for he adds "good putty no back putty." In 1774 Stephen Moore protests against his displacement by Thos. Ivory in the building operations.

### The Pride of Place.

In 1775 difficulty arose with the London firm of Thos. English and Sons over the width of the iron gates to be supplied, the measure in dispute being three inches. The contractors seem to have pacified the Governors by stating that if St. Thomas' Hospital, in the High Street, Borough—a much frequented thoroughfare—was content with gates 10 feet wide, surely Norwich, with its more spacious approach, might be satisfied with 10 ft. 9 in. instead of 11 ft., and in the end it was satisfied.

### Dinners and Sermons.

In these early days the institution received substantial financial support from hospital sermons, plus the dinners which followed the sermons at inns like the Maid's Head and the White Swan. As much as £143 was collected from such sources in 1779, increasing to £230 in 1806. The *Norfolk Chronicle* of 1808 reports the inclusion of 26s. 6d. base money taken at the collection. Was its inclusion intentional or accidental? Have we not experience of buttons being offered in response to appeals for financial assistance?

### Music a Disappointment.

The hospital's early association with our musical festivals is not without a touch of humour. The receipts of the first festival amounted to £2,399; in 1827 to £1,673. Such results were too good not to create a desire on the part of smaller local institutions to share in them, and an appeal to attain that end was made, to meet with an immediate refusal for the time being, but a promise was given that one-quarter of the receipts of the 1833 festival "should be divided equally between the Blind Institution, the Eye Infirmary, and the Norwich Dispensary." To them the future would be big with promise, but alas, the net receipts of that year were but £36, and the expectations of these smaller institutions were grievously disappointed. In 1839 it is recorded that the festival resulted in a deficiency of £231, and it was then resolved that in future the funds of the hospital should not be used for the purpose of the festival, a resolution resulting in the creation of the Guarantee Fund now associated with every festival effort. History is prone to repeat itself.

### From Bread to Potatoes.

In 1800 was issued a Royal Proclamation against waste, and the dietary of the hospital was changed from bread to potatoes. In this year of grace 1916 his Majesty's Government is pressing on all sections of the community the necessity for economy, and the avoidance of unnecessary waste. It would be gratifying to the Governors of those early days to learn (I quote from a newspaper of 1805) that when Mr. James Neild, the philanthropist, visited the hospital, he left on record the opinion that "it does honour to the country and is one of the best I have seen."

### Tracts and Drums.

It was a practice common in those days to supply tracts to each departing patient, but by the changes made in the character of the tract I fear the good intentions of the donors did not always secure an appreciative response on the part of the patient. The degree of appreciation is not always commensurate with the cost of their production. In the early part of the last century the hospital had among its movable assets drums. They were borrowed for musical purposes, and in 1810 were loaned to Cambridge for the Festival then to be held in that town. Can it be supposed that the staff of the hospital was sufficiently strong to create out of its members a band? I have no explanation to account for their possession; only a latent suspicion that those instruments were used for other purposes. The *Norfolk Chronicle* of January 1847 records that the use of ethereal fumes had been introduced into the hospital by Dr. Hull in the extraction of teeth. The same journal in December of the same year reports the first use of chloroform in an operation upon a young woman undergoing amputation. It states that she became insensible to pain, and that her sensations were apparently of a happy description, for she not only sang psalms, but held lively conversations with imaginary persons.

### A Preference for Old Ways.

Clearly the hospital had then entered upon a new era of usefulness, but new methods were, I fear, not viewed with favour by the members of the old school. Anyhow that year (1847) saw disunion among the staff, a report being presented to the governors by certain members, in which it was alleged "that compared with other hospitals of the same size the utility of the Norfolk and Norwich Hospital was in an inverse ratio to its cost of maintenance." I have read the newspaper records of the period, and the correspondence which followed, and come to the conclusion that preference for old ways and methods was responsible for the charges, though defects in administration were admitted. It is but human nature that the older we get the stronger becomes our allegiance to the things that are or have been. The dispute ended in the passing of a resolution "to improve the management of that noble building." Gentlemen, the past and its relations to the present constitute ever a fruitful study. It is good to follow the evolution of a great institution, and to realise how many of the privileges we enjoy to-day are the result of patient labour of the generations that have preceded our own.

With the sources of Mr. Southwell's information and the inspiration of his example we deal in a Note this week.

